# The Treatment of Pneumonia in Guy’s Hospital

**Published:** 1843-05

**Authors:** 


*The Treatment of Pneumonia in Guy's Hospital.—The pure Antimonial cure.*—No one mode of treatment should be adopted in pneumonia. To say that venesection, twice or thrice repeated, or antimony, or calomel and opium, should always be the remedy, would be dangerous in practice. If the disease existed always in persons of the same habits, country, and locality; was always presented in the same stage, uncomplicated (and so on), then one treatment might be adopted with advantage; and tables, showing the cures and deaths, from one or the other mode, might be trusted as guides. But each case should be more or less studied by itself. I shall presently have to recommend moderate venesection, followed by calomel, opium, and antimony; but a young man was admitted into Guy's hospital a few weeks ago suffering from pneumonia, passing to the second stage; no indication of tubercles could be discovered; he had no typhoid symptoms; but he was so feeble that bleeding would have killed him, or antimony would, I believe, have extinguished life. He was treated for the first twenty-four hours with ammonia; after which he was ordered in a pill,—two grains of blue-pill, two of extract of hyoscyamous, and one of ipecacuanha,—and a draught composed of ten minims of liquor potassæ and an ounce and a half of decoction of bark, every six hours; a blister to the side, and mild nutritious diet. It is true that a few days after, when he had improved in power, he was put upon calomel and opium, and antimony; but these were withdrawn in two days. He then had merely a little saline medicine, followed by quinine; blisters to the affected side were several times repeated during the period, and he left the hospital in a few weeks, cured of his inflammatory complaint, though still weak. He will probably ultimately die of phthisis.

In the average of cases in this hospital a considerably larger proportion of those who were bled recovered, than of those who were not. But that venesection is therefore advantageous in pneumonia, abstractedly considered, could not be fairly supported, as venesection was, I believe, practised in *all cases*, excepting in those who were already too much debilitated, or too slighly affected.

The plan for many years generally adopted in Guy's Hospital, in acute pneumonia, has been to bleed the patient to approaching syncope, and to administer a pill, containing half a grain of opium, and a quarter of a grain of tartarised antimony with one or two grains of calomel, every three, four, or six hours, according to the severity of the symptoms, usually combined with a saline mixture, containing twenty or thirty minims of antimonial wine. If, in a few hours, or next day, the general symptoms have been unsubdued, or have returned, venesection has been repeated. It has sometimes been necessary to bleed again and again. Triple venesections have been uncommon, and a fourth very rare. If the general symptoms, on the contrary, have been reduced, though the local affection has continued severe, or if the patient's power has been materially diminished by venesection, cupping has been ordered, to from six to twelve ounces. On a decrease of the disease, the medicines have been less frequently repeated, or stopped, even though the mercury has not affected the mouth. If the system has evidently become affected thereby, but the complaint is still active, it has usually been discontinued, or repeated only in small unfrequent doses. Blisters also have been applied with good effect in the latter stages. The utility of the latter remedies has been doubted; but I state my thorough conviction of benefit being derived therefrom, in very many instances. The foregoing treatment has been, on the whole, so efficient that no important change has been considered justifiable.

I have frequently been desirous of trying the pure antimonial, or contra-stimulant, treatment; but in so important a disease I have not felt justified in discarding means which have so often effected a cure. I must, however, confess that the results of the treatment of pneumonia in Guy's Hospital—if all cases, however advanced, and however complicated, be taken into account—are not to be compared with what are stated to have been the "triumphant" effects of antimony. Thus Laennec says, that of 62 cases treated by antimony, only 6 died, two being moribund on admission, two old men of seventy, with cerebral congestion and pleurisy, and the sixth under disease of the heart. Others are reported to have lost only 1 in 30 and 1 in 40 cases. However, my observation is entirely opposed to Laennec, when he states that he has never known the disease renewed when antimony had effected amelioration. In the Infirmary of Edinbnrgh, while I was a pupil there, a patient after two venesections, leeches, and continued antimony, was considered almost convalescent. The antimonial solution was however continued. But when the disease had for four days appeared to be rapidly decreasing, the attack was renewed; and it was necessary again to bleed him to ten ounces, after which he rapidly recovered. The same has certainly happened in several cases that have fallen under my notice. In some instances, also, when antimony has been at first employed with benefit, but relapses have taken place, it has appeared necessary for the cure to administer calomel and opium in combination with it. Nevertheless, I believe antimony to be especially useful in persons who cannot bear the abstraction of blood, and those in which mercury is contra-indicated. In such cases, as in the pneumonia and bronchitis of children after measles, I have occasionally administered it, and with manifest advantage. The patients have, it is true, died from another complaint; but that complaint has been, I have thought, produced by the remedy employed for the pneumonia.

The great authority last quoted considers antimony to be equally efficacious "in any stage of the disease, even after a great portion of the lung has undergone purulent infiltration;" and supposes that it may act by "increasing the activity of *interstitial* absorption." But the well-known action of mercury in promoting the absorption of effused matter, clearly points it out as the medicine especially indicated when there is inflammatory deposit.

From the whole circumstances, I believe that antimony is a very active remedy in pneumonia, more particularly indicated in slight and recent cases, those complicated with bronchitis, and those in which venesection cannot be borne or repeated, and mercury cannot be safely employed; that the cases are often more rapidly relieved by it than by any other means, but that they are also more than ordinarily liable to relapses; and that when consolidation has obviously occurred, it should not be trusted alone, but should always be given with mercury.—*Dr. Hughes, Guy's Hosp. Rept.*, October.—*Lancet.*

